# Impact of fog on California waterfowl flight activity: historical and modern insights into effects post-Clean Air Act

**DOI:** 10.1007/s11356-026-37659-2

**Published:** 2026-03-30

**Authors:** Fiona McDuie, Cory T. Overton, Austen A. Lorenz, J. Patrick Donnelly, Desmond A. Mackell, Elliott L. Matchett, Mark Petrie, Michael L. Casazza

**Affiliations:** 1 U.S. Geological Survey, Western Ecological Research Center, Dixon Field Station, 800 Business Park Drive, Suite D, Dixon, CA 95620 USA; 2 San Jose State University Research Foundation, Moss Landing Marine Laboratories, 8272 Moss Landing Rd, Moss Landing, CA 95039 USA; 3 US Fish & Wildlife Service, Intermountain West Joint Venture, Migratory Bird Program, 1001 S. Higgins Avenue, Suite A1, Missoula, MT 59801 USA; 4 Ducks Unlimited, 3074 Gold Canal Drive, Rancho Cordova, CA 95670 USA

**Keywords:** Radiation fog, Tule fog, Flight, Energetics, Pollution, Clean Air Act, Waterfowl

## Abstract

Since establishment of the Clean Air Act in the early 1970s, occurrence of the dense ‘Tule Fog’, historically prevalent throughout winter across California’s Central Valley, has substantially reduced. At the same time, waterfowl body masses have generally increased. Flight is metabolically expensive, and fog visually and navigationally impairs birds in flight, likely causing them to remain aloft for longer than usual periods. If less fog results in less flight and reduced energy expenditure, then fewer winter Tule fog events could contribute to increased body masses of California waterfowl since the mid-1980’s. Therefore, we aimed to assess the relationship between waterfowl flight and fog occurrence/density with historic (1991–93) and modern (2015–23) waterfowl tracking data in the Central Valley of California (CCV). Historic tracking data showed that the probability of flight increased with increasing fog density. Birds were significantly more likely to fly in fog than when there was no fog and most likely to fly in heavy fog*.* Modern data showed similar responses to fog with flight occurring significantly more during dawn fog events. This relationship between improved waterfowl body mass and fewer fog events may provide an opportunity to redirect scarce funding to focus on other population requirements such as improving habitats for nesting, molting and brood rearing that are currently lacking. Unanticipated benefits of the Clean Air Act should stand as a strong recommendation to maintaining this Act into the future.

## Introduction

One of the most impactful environmental legislations in the United States has been the Clean Air Act (CAA), implemented by the Environmental Protection Agency (EPA) in 1972 (U.S. Environmental Protection Agency 1972). This comprehensive public health and environmental protection initiative, enacted to reduce concentrations of air pollutants (see EPA’s Air Trends site; Gray et al. 2019), has had substantial positive impact on public health and welfare (Ross et al. 2012; Nethery et al. 2021). Another act which contributed to reducing impactful air pollution was the Rice Straw Burning Reduction Act (1991). Rice is an important agricultural crop in California’s Central Valley (CCV) and Sacramento Valley (Central Valley Joint Venture 2020), and this act was established to reduce pollution generated by the traditional post-harvest burning of rice straw. Contemporaneously, the occurrence of the heavy, dense, low-lying fog, influenced by air pollutants and known as ‘tule fog’ in the CCV, has significantly reduced (76%) since the early 1980’s (Baldocchi and Waller 2014; Gray et al. 2019). Tule fog historically shrouded the CCV regularly during the wet winters and could extend over 400 miles from Bakersfield in the south to Red Bluff in the north of California. However, consistency between less frequent tule fog and declining air pollutants indicates that cleaner air drives reduced fog occurrence (Baldocchi and Waller 2014; Herckes et al. 2015, Gray et al. 2019). Gray et al. (2019) demonstrated that California has experienced 5 fewer foggy days per year for every decrease of 10 ppb of oxides of nitrogen in the past 36 years.

The goal of this study is to understand how the reduction in fog has impacted waterfowl flight activity, which may ultimately affect energetic needs for waterfowl in CCV. The CCV, hosts one of the largest global concentrations of migratory birds during the fall and winter and is vital to North American waterfowl populations (Bellrose and Kortright 1976; Central Valley Joint Venture 2020). For these wintering birds, heavy fog obscures visibility and interferes with orientation, navigation and migration (Alerstam 1990; Newton 2007; Kirsch et al. 2015; Becciu et al. 2021). When the ground is concealed from airborne birds, they are less able to detect suitable habitat or safe refuge and are unable to land. Therefore, heavy fog may cause birds to spend more time flying or fly out of fog to more distant roost or feeding locations. Amplifying this effect, is pressure from hunting during the annual waterfowl hunting season, which disturbs waterfowl, causing them to fly more often and for longer periods (McDuie et al. 2021). Flight is one of the most metabolically demanding activities a bird can engage in, with metabolic rates often exceeding 10 times a bird’s resting metabolic rate (Schmidt-Nielsen 1972; Wooley and Owen 1978; Nudds and Bryant 2000). Increased flight during heavy fog events would cumulatively increase energy expenditure, resulting in reduced body condition if not compensated for by increased food intake. Conversely, if tule fog occurs with less frequency, flight and energy expenditure may be reduced resulting in improved physical condition.

Energy consumption and expenditure are important considerations for wintering waterfowl populations, and broad-scale waterfowl management actions by the Central Valley Joint Venture (CVJV) in the CCV since 1990 have successfully focused on ensuring sufficient energy to sustain wintering waterfowl populations (Central Valley Joint Venture 2020). Fleskes et al. (2016) attributed improved average waterfowl body masses in the mid-2000s since the early 1980 s to the improved food conditions for waterfowl in the CCV (Central Valley Joint Venture 2020). Interestingly, these waterfowl body condition improvements occurred over the same approximate timeframe since implementation of the CAA and the associated concurrent fog declines.

While there is no doubt that energy and food management actions have significantly benefited wintering waterfowl populations, it is possible that the declines in tule fog may have resulted in reduced flight, and consequently, lower energy demand, which may theoretically explain why waterfowl body condition has improved in recent decades. Fog impacts on waterfowl during winter have not been studied, but these large overwintering CCV waterfowl populations provide an opportunity to investigate this possibility. Potential, unanticipated benefits to waterfowl, may afford an opportunity to reconsider food-focused management plans, and redirect future efforts to other priority objectives such as essential habitats that are currently lacking. Therefore, our study seeks to determine whether observed body condition improvements in these populations may be attributable, at least partly, to reduced Tule fog occurrence in the CCV. Such a systemic change to energy demand in the CCV could result in changes to limiting characteristics of the landscape and provide managers with the opportunity to focus initiatives toward foraging needs that are less well understood, such as prey-switching to invertebrates late in the winter, or to other priority objectives such as essential non-wintering habitat needs such as nesting and molting, which are currently lacking (Central Valley Joint Venture 2020). Our study evaluates whether tule fog influences the amount of time birds spent in flight in two different time periods – ‘historic’ (telemetry waterfowl tracking data from 1991–93), and ‘modern’ (GPS waterfowl tracking data from 2015–23), by analyzing the probability of flight in each period. In the modern dataset we will also assess how the probability of flight differs according to time of day (dawn/dusk).

## Methods

### Field methods

We conducted our study using waterfowl tracking data collected by radio telemetry (1991–93) and GSM-GPS (2015–23) in the CCV. We captured birds using standard techniques, with rocket nets and baited funnel traps (Schemnitz et al. 2009), and all were released at the location of capture after ~ 20–30 min handling.

For the historic study 1991–93 (Casazza et al. 2012) we captured Northern pintails (*Anas acuta*) on Grizzly Island Wildlife Area (GIWA) and Suisun Marsh during late August and early September, We deployed 102 and 101 backpack-style VHF radio transmitters on pintails in 1991 and 1992 respectively (Casazza et al. 2012), using backpack harnesses constructed according to methods described in Dwyer (1972). VHF devices weighed 18 g (approximately 2% of body weight) and included motion sensitive mortality switches (Advanced Telemetry Systems of Isanti, Minnesota, USA; Casazza et al. 2012).

In the 2015–23 study, we captured Northern pintail in Suisun Marsh, GIWA and the Sacramento Valley and mounted solar powered, remotely programmable GPS-GSM transmitter units by 1) Ornitela®: OrniTrack-15 (58 × 25 × 14 mm, 15 g; N = 151) or OrniTrack-10 (47 × 18 × 12 mm, 10 g; N = 25), and 2) Ecotone® (Gdynia, Poland) GPS-GSM Saker L (58 × 27 × 18 mm, 17 g; N = 74) or Crex-XS (36 × 25 × 19 mm, 14 g; N = 31). We mounted GPS-GSM on small foam pads with epoxy glue and attached to duck’s backs with a backpack harness constructed of 5 mm nylon-neoprene knitted elastic ribbon, which minimizes wicking of moisture to down feathers while allowing normal movement and flight (< 3% body weight; McDuie et al. 2019). GPS-GSM devices store data onboard for location (latitude, longitude), and date-time, until transmitted via GSM cellular text message to manufacturer-operated servers (Ornitela or Ecotone), with sufficient battery power and GSM signal strength. Data are transferred to the Movebank online platform for storage and access (Kays et al. 2022; Kranstauber et al. 2011; Wikelski et al. 2022). We marked all birds with individual identifying leg bands (USGS Bird Banding Lab; Smith 2013).

### Measuring flight activity relative to fog occurrence

#### ‘Historic’ bird flight activity

Historic Northern pintail flight activity was assessed from 114 daily summarized field observations recorded while waterfowl were tracked between 11/7/1991 through 3/1/1993. We attempted to relocate pintails remaining in the primary study area of Suisun Marsh ≥ 5 times per week during daylight (relocations: N = 4,409, individuals: N = 201) and nighttime (relocations: N = 3,279, individuals: N = 165) using radio-telemetry (Casazza et al. 2012). The number of individuals tracked varied from one to 53 birds per date. We determined all pintail locations by keying vehicle alignment azimuth, location (Universal Transversal Mercator (UTM) units) and bird azimuths into a modified version of the XYLOG and UTMTEL programs (Dodge and Steiner 1986). By minimizing time spent traveling between locations we obtained two or more azimuths at approximately 90° angles from the pintail location, helping prevent location bias (Schmutz and White 1990). We did not approach pintails within 100 m and avoided measurements > 1.5 km away from actual pintail locations. The average error distance to test transmitters for our telemetry system was 58 ± 35 m (Casazza et al. 2012). Individuals in flight were identified during vehicle-based triangulation. We tracked each bird in flight by recording bird azimuth from a fixed location using a truck mounted dual yaggi telemetry system. If the azimuth changed during that observation (usually more than 5 degrees) over the course of a few minutes, then birds were recorded in field records as "flying". We recorded the proportion of birds recorded in the field records as "in flight" and determined the proportion as a ratio of birds observed that day flying/total observed (Casazza et al. 2012).

Local fog conditions were recorded during field tracking and categorized into four classes reflecting decreasing visibility: no fog, light, moderate, and heavy fog. We used logistic regression to estimate the probability of an individual being in flight during historic tracking periods for each category of fog occurrence and controlled for intra-annual variation in flight activity using a random effect reflecting the calendar date. The log-odds of being in flight were contrasted across fog density categories with a Tukey adjustment for multiple comparisons among four groups. Analysis was performed using the `glmer` function within the “lme4” package (Bates et al. 2015), and contrasts were performed using the `emmeans` and `pairs` functions in the “emmeans” package (Lenth 2022). We used R software version 4.1.3 (R Core Team 2022) running in R studio version 2022.07.1 ©RStudio, PBC for all analyses.

#### ‘Modern’ bird flight activity

**Fig. 1:**
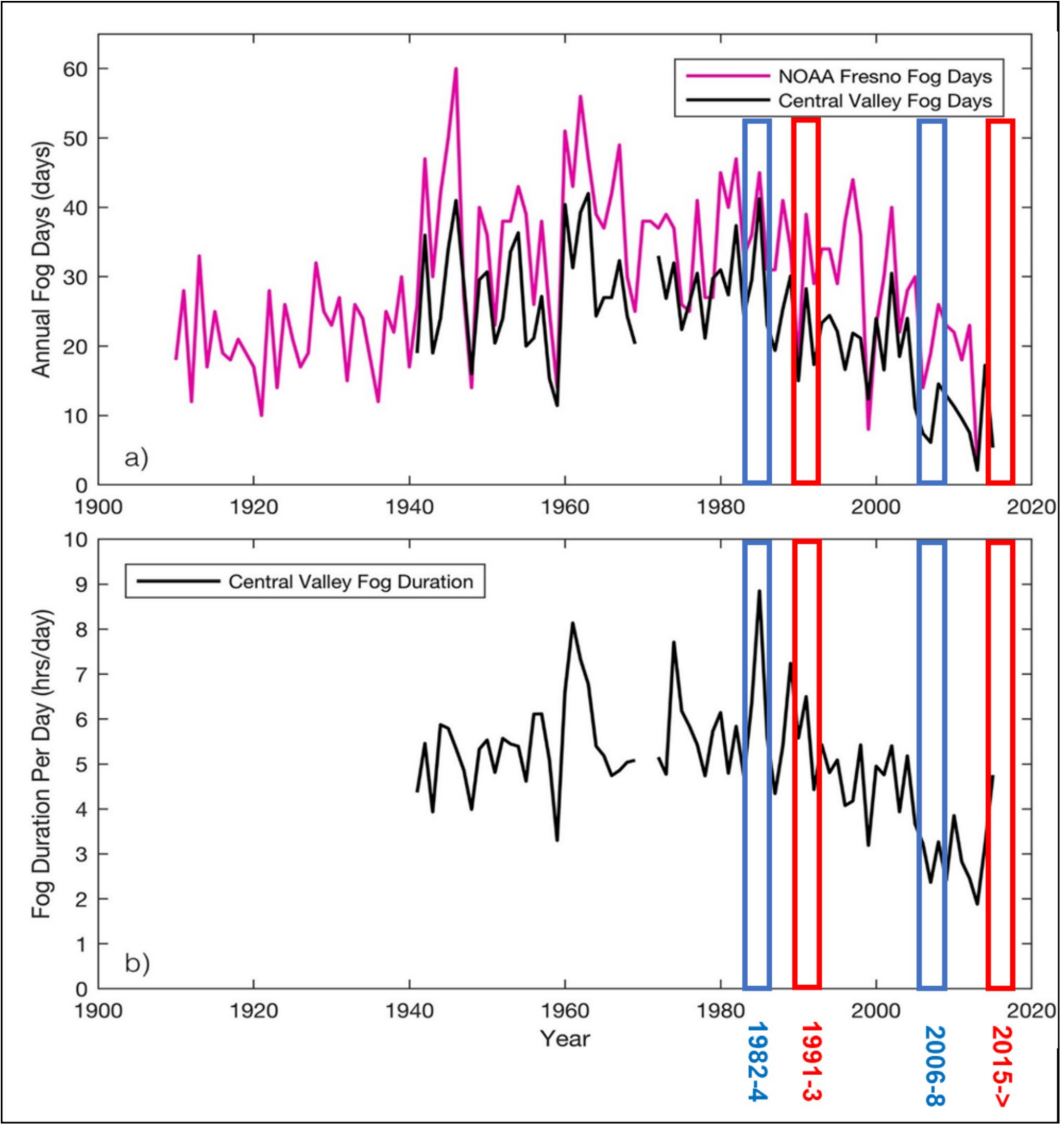
Adapted from Gray et al. (2019) showing reductions in tule fog occurrence and duration in California’s Central Valley. Blue boxes indicate the years that waterfowl body conditions were measured by Fleskes et al. (2016: 1982–4 and 2006–8) and red boxes the years that telemetry (Casazza et al. 2012; 1991–93) and GSM-GPS (2015–23) data were collected for flight analysis

**Fig. 2:**
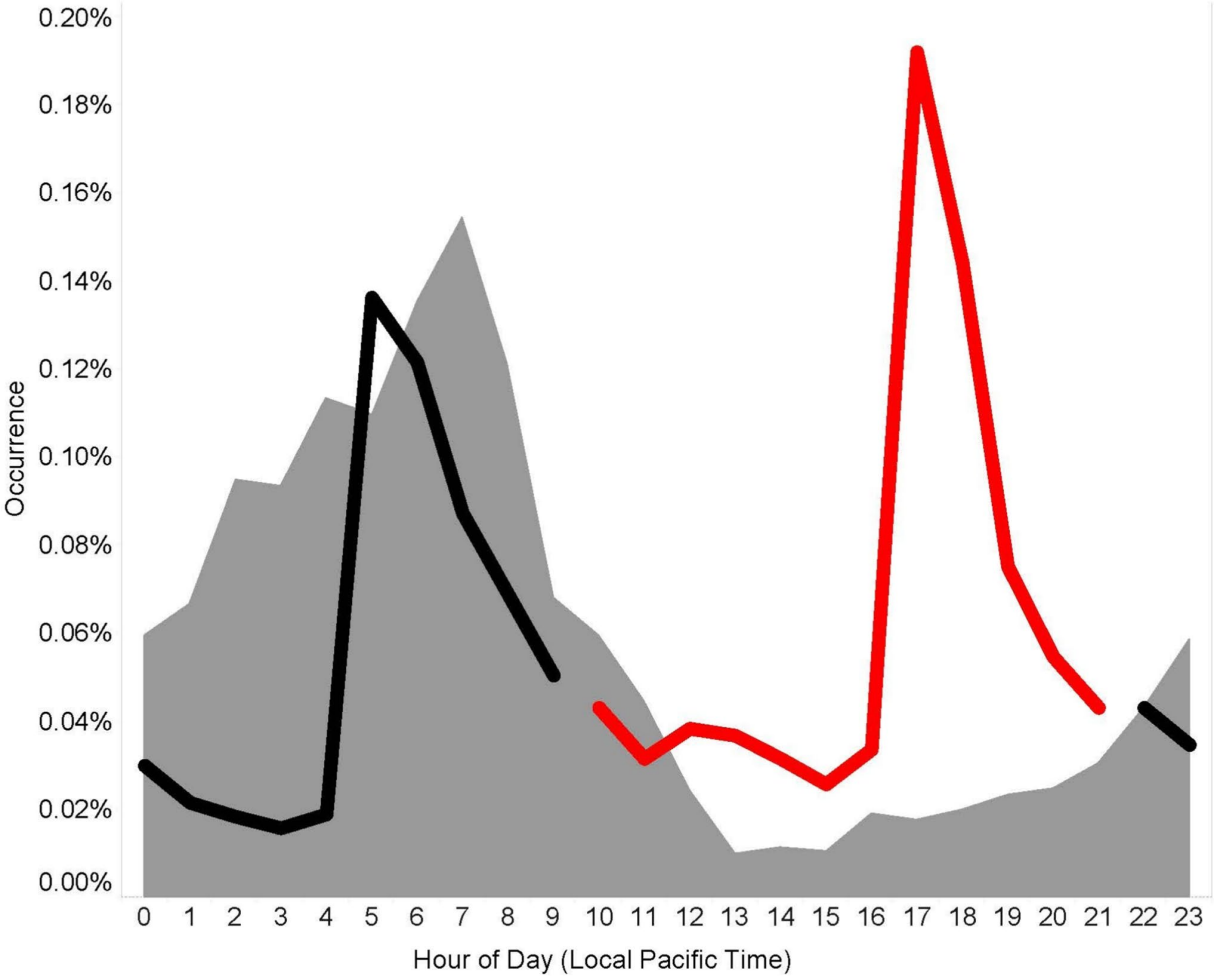
Modern (2015–23) foggy periods (gray areas) in the Central Valley have generally been restricted to overnight and early morning periods. Duck flight activity (all locations) to and from foraging and roosting areas occurs during both dawn (black line) and dusk flight periods (red line) respectively, but fog was more likely to coincide with dawn foraging flight

All available GSM-GPS tracking data for Northern pintail occupying the CCV between 10/1/2015 and 11/28/2023 (n = 684,970 locations) were downloaded from Movebank (Wikelski et al. 2022). We filtered available locations to the 141,166 locations occurring within 16.1 km (10 miles) of an airport with recorded METAR-formatted weather information available through the “pmetar” package in R (Cwiek 2023). The most recent recorded weather information for the closest airport was used to annotate the visibility range at each duck location. Weather information was recorded at intervals ranging from 5 min to every 20 min among the 16 airports providing data; therefore, we assume the METAR weather data from the closest airport is both spatially and temporally equivalent to conditions at the associated bird location. The presence of fog in the weather description or the identification of visibility less than 403 m (0.25 miles) indicated foggy periods used in subsequent analyses. We determined that telemetry locations reflected flight when ground speed estimates obtained during GPS coordinate calculation exceeded 5.75 km per hour. We used mixed logistic regression to estimate the probability of flight during fog versus periods without fog while using a random intercept for each hour of the day to account for circadian patterns in activity (McDuie et al. 2019) and a random intercept for day of season to account for variation across the winter. Due to the decreasing occurrence of winter Tule fog (Fig. 1), continuous periods of dense fog have been less common and generally did not occur during more recent GPS telemetry seasons. In recent years, fog has been occurring primarily in the morning, whereas waterfowl still fly during each crepuscular period as they search for food (McDuie et al. 2019). Therefore, we compared models of flight occurrence during periods where fog generally occurred (10 pm to 10 am: “dawn” flight period) to models including periods where fog was less frequent (10 am to 10 pm PT: “dusk” flight period; Fig. 2).

## Results

### Historic bird flight activity

**Fig. 3:**
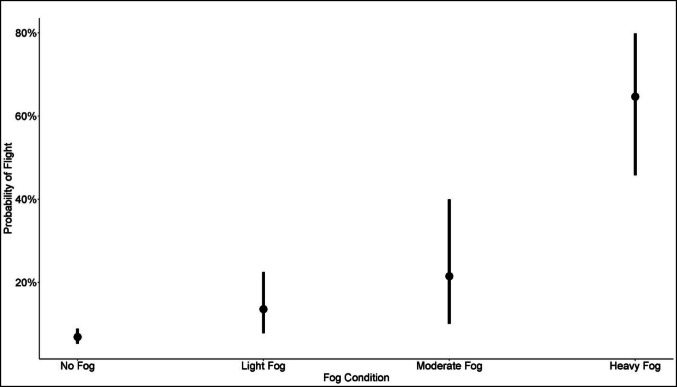
Probability of waterfowl being in flight during 1991–93 vehicle-based telemetry increased as fog was assessed to be denser shown with 95% CI error bars

Historically, the probability that individual tracked waterfowl were flying during vehicle-based telemetry increased with increased fog density (Fig. 3, Table 1). The probability that an individual tracked waterfowl was flying in heavy fog was 9.4 times (64.7%) the probability that an individual was flying when no fog was present (6.9%). When contrasting the probability of flight relative to changes in fog density, birds were significantly more likely to be flying when fog was moderate or heavy compared to when there was no fog, and when fog was heavy compared with moderate or light fog (Table 2).

**Table 1:** Mixed-logistic regression indicated waterfowl increased flight activity during historic periods of fog and that denser fog was associated with a greater probability of being in flight during vehicle-based telemetry

Predictors	Log-odds	CI	*p*
fogfactor (no fog)	− 2.61	− 2.88 to − 2.33	< 0.001
fogfactor (light fog)	− 1.85	− 2.46 to − 1.24	< 0.001
fogfactor (moderate fog)	− 1.30	− 2.18 to − 0.41	0.004
fogfactor (heavy fog)	0.60	− 0.17 to 1.38	0.125
Random effects			
*σ*^2^	3.29		
^*τ*^00 DayofSeasonNov1	0.59		
ICC	0.15		
^*N*^ DayofSeasonNov1	92		
Observations	114		
Marginal *R*^2^/conditional *R*^2^	0.116/0.250

**Table 2:** Contrasts of the probability (log-odds) that waterfowl were in flight during vehicle-based telemetry among categories of fog density identified during telemetry activities (1991–93)

Contrast	Estimate	SE	*z* ratio	*p* value
No fog—light fog	− 0.754	0.327	− 2.307	0.0963
No fog—moderate fog	− 1.31	0.461	− 2.84	0.0234
No fog—heavy fog	− 3.209	0.421	− 7.626	< 0.0001
Light fog—moderate fog	− 0.556	0.55	− 1.01	0.7435
Light fog—heavy fog	− 2.455	0.487	− 5.044	< 0.0001
Moderate fog—heavy fog	− 1.899	0.6	− 3.168	0.0084

### ‘Modern’ bird flight activity

**Fig. 4:**
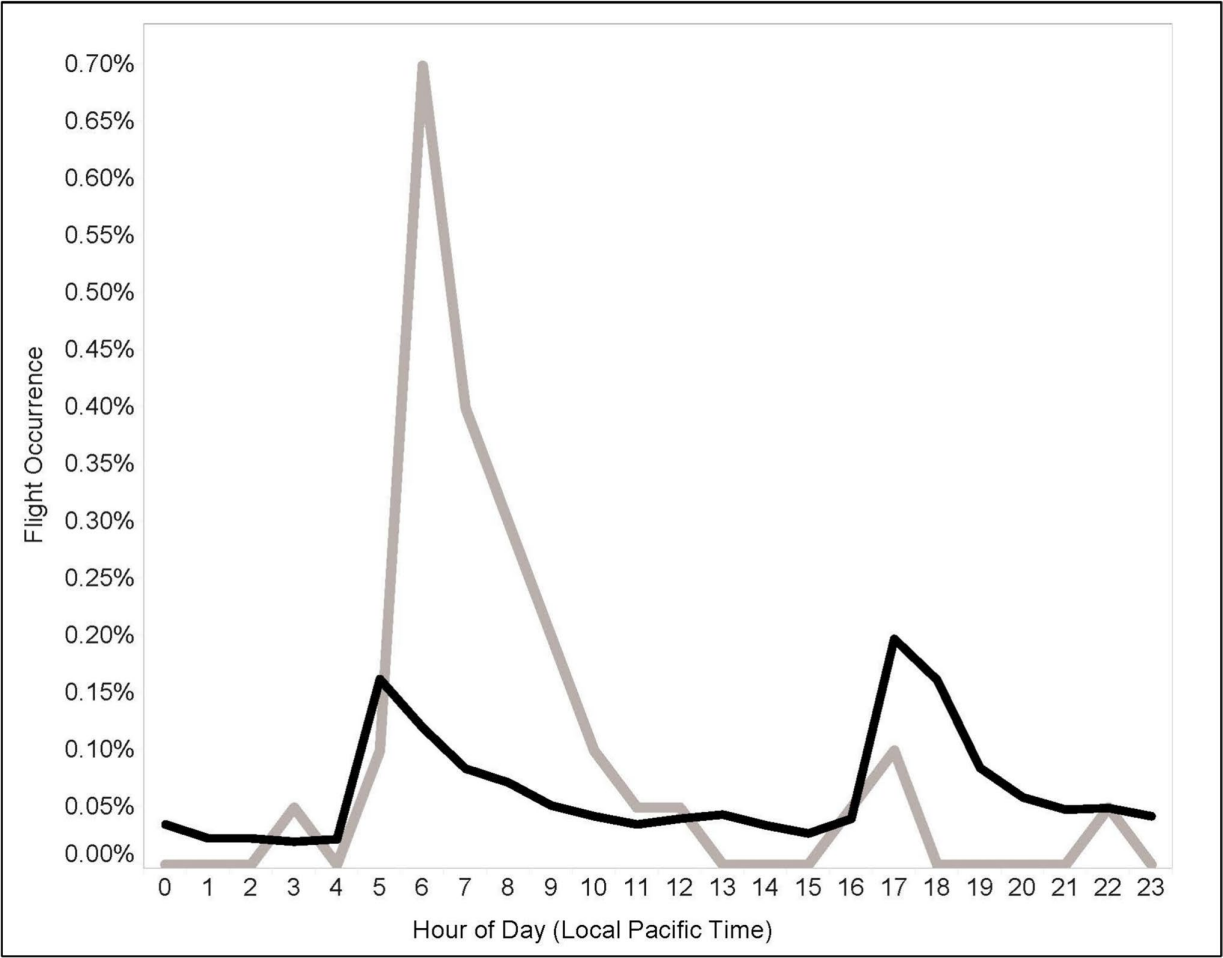
The occurrence of modern (2015–23) flight during periods without fog (black line) followed a pattern of elevated but approximately equal flight activity during dawn and dusk periods, indicating natural biorhythms under non-foggy conditions. Flying during periods with fog (grey line) was observed significantly more often during dawn periods with fog, but not during dusk flight periods with fog. Historic (1991–93) tracking was not resolved to time of day so this figure represents only the modern data

Of the 141,166 pintail locations occurring within the observation range (16.1 km) of METAR reporting airports in the Central Valley of California from 2015 through 2023, 1.42% occurred during periods of fog and 1.55% were locations obtained while birds were flying. Across the entire day, there was limited evidence that flying increased during fog (Odds-ratio of flight = 1.32; 95% CI: 0.95–1.84; p = 0.100). During the dusk flight period (when fog was less common, there was no evidence that flight was more prevalent during fog (Odds-ratio of flight = 0.90; 95% CI: 0.35–2.30; p = 0.825). However, during the dawn flight periods flying was significantly more common during fog (Odds-ratio of flight = 1.53; 95% CI: 1.07–2.19; p = 0.020; Fig. 4).

## Discussion

Our research finding that Northern pintail flew more during fog events suggests California waterfowl are likely one of the beneficiaries of improved air quality that has resulted from implementation of the federal Clean Air Act (U.S. Environmental Protection Agency 1972). Propensity to fly was particularly impacted during heavy fog events and, in the modern tracking period, this was more apparent during flight at dawn than dusk. Over the decades since establishment of broadscale anti-pollution actions, and consequent reductions in fog events across the CCV (Gray et al. 2019), large migratory overwintering populations (and even resident species) of California waterfowl demonstrated improvements in their physical condition (Fleskes et al. 2016). Those authors reasoned that these improvements were related to management actions focused on providing food resources in the CCV. CVJV planning is aimed primarily at ensuring sufficient energy is provided on the landscape to support population targets for overwintering waterfowl (Central Valley Joint Venture 2020). Actions include post-harvest flooding of rice fields, which provides habitat and waste grain, and encouraging growth of waterfowl friendly seed crops in private properties such as duck hunting clubs. These actions have driven substantial improvements in habitat and targeted food growth in the CCV, which has almost certainly contributed to observed improvements in waterfowl body condition (Fleskes et al. 2016). However, we argue that these improvements may also be driven, at least partially, by reduced waterfowl flight times, and associated reduced energy expenditure, due to decadal reductions in dense fog occurrence driven by cleaner air, and our findings support this theory.

Estimates of energy requirements from available CCV habitat, upon which management practices are based, imply that there is potential for energy deficit during late the winter period (Central Valley Joint Venture 2020), which would negatively impact migration (Alerstam 1990; Newton 2007; Kirsch et al. 2015; Becciu et al. 2021). However, these estimates are based on waterfowl energetics that have not accounted for the influence of fog on probability of flight. If ducks are expending substantially less energy over the winter due to less fog, this has implications for waterfowl ecology, management, and conservation across the region. With less energy required, waterfowl would obtain and retain even greater energy from currently available food resources, consequently reducing the amount of habitat (equates to energy, based on CVJV planning) needed to support population targets. This would have the effect of increasing population carrying capacity and should improve CCV waterfowl population trajectories.

Within the CCV, waterfowl habitat requirements are estimated using the TRUEMET bioenergetics model (Petrie et al. 2016) adopted in 2019. This model compares assumed food energy needs with food energy supplies, to define habitat objectives and conservation goals in the CCV. Using TRUEMET, the CVJV defined long-term waterfowl targets by modeling the total area of habitat required to support waterfowl populations under those energetic conditions (Central Valley Joint Venture 2020). If waterfowl populations do readily obtain sufficient energy from current habitat, as suggested by improved body condition observed by Fleskes et al. (2016), TRUEMET could potentially adjust energetic estimations, resulting in potential lowering of needed habitat and food requirement estimates. Moreover, waterfowl condition may continue to improve if fog occurrence continues to decrease, further feeding back into TRUEMET estimates. This may create opportunities to instead examine conservation practices that accommodate nesting, brooding and molting activities, which have been identified as lacking (Feldheim et al. 2018; Kahara et al. 2022), and are currently classed as lower priorities by the CVJV (2020). Such efforts could support a wider network of protected wetland habitat mosaic with adequate sanctuary and upland resources and perhaps allow for higher population targets. Incorporating changes in waterfowl body condition and energetic needs into TRUEMET might offer a different perspective on current habitat condition and future food requirements of waterfowl populations under various land use changes and drought scenarios (Petrie et al. 2016).

Declines in specific atmospheric pollutants and concomitant reduction in fog occurrence with CCV (Gray et al. 2019) may be producing longer-term, lasting benefits for waterfowl. Air pollution has been associated with bird health, abundance, and activity declines across the U.S.A. (Nihei et al. 2024). One study indicated 40-year improvements to air quality may have averted the loss of ~ 1.5 billion birds (Liang et al. 2020). Migration movements are negatively affected by the presence of fog (Alerstam 1990; Newton 2007; Kirsch et al. 2015; Becciu et al. 2021), and many species initiate their northbound migration from the CCV in late winter, at a time when their progress would be affected by dense fog events. However, fewer fog days and associated improved body mass could contribute to more efficient northbound migration in the spring. Additionally, body condition in geese and other waterfowl species has been shown to have significant cross-seasonal effects on subsequent reproductive output (Legagneux et al. 2012; Clausen et al. 2015; Zarzycki 2017), so reduced energy expenditure during foggy winters could have beneficial carryover effects into the summer breeding season.

There are several scenarios in which an improved understanding of energetic capacity in the Central Valley would enhance the adaptability of conservation planning (e.g., Central Valley Joint Venture, BirdReturns (Reiter et al. 2018) and other incentive programs). For example, climate-driven wetland drying recently documented in the Pacific Flyway raises concerns over the emergence of new and powerful resource bottlenecks in waterfowl habitat networks (Donnelly et al. 2022). Landscape change can expose waterfowl populations to multiple independent risks due to life history strategies supported by diffuse regions separated by hundreds or thousands of kilometers (Zurell et al. 2018). Risks are compounded by cross-seasonal effects where environmental conditions experienced in one location (breeding grounds, wintering grounds or stopover areas) can affect the fitness in subsequent locations leading to declines in long-term demographic performance (Sedinger and Alisauskas 2014). Therefore, identifying and prioritizing actions that offer beneficial, multisectoral solutions may be crucial in protecting wildlife in the long term.

## Conclusion

The study reveals that the flight activity of California waterfowl, specifically Northern pintails, is significantly influenced by fog density. Our findings suggest that improved air quality since the implementation of the Clean Air Act, which may have led to a reduction in fog occurrence (Gray et al. 2019), could, in turn, decrease energy expenditure for waterfowl during winter due to fewer foggy days. This ripple effect may be sufficient to change the energy balance of waterfowl in portions of the CCV where fog used to occur more frequently, and could inform adaptive management, resource planning, and optimal conservation interventions (Hopkins et al. 2022). Factors that have reduced the energy demand of waterfowl, intended or not, could support more effective waterfowl conservation and resource management. This highlights the importance of looking beyond the intended scope of broadscale environmental regulations such as the Clean Air Act to understand potential implications to wildlife.

## Data Availability

All data used for this study are stored online at USGS ScienceBase—Overton, C.T., and Casazza, M.L., 2025, Waterfowl tracking VHF and GPS data relative to Sacramento Delta Fog and Clean Air Act: U.S. Geological Survey data release, 10.5066/P14UBHN2. The modern data are available in the Movebank online platform for storage and access (Kranstauber et al. 2011; Kays et al. 2022; Wikelski et al. 2022).
